# Arthrectomia Talocruralis

**Published:** 1889-04

**Authors:** Marcell Hartwig


### *ARTHRECTOMIA TALOCRURALIS.*

For extirpating the synovial membrane of the ankle-joint without injury to the bones, although the latter can be taken out, partly or wholly, according to the extent of the disease, Meinhardt Schmidt, *Centralblatt für Chir.*, February, 1889, recommends a longitudinal incision in front and one in the rear of the joint. The latter falls a little to the outside of the median line, the tendo Achillis is drawn inwards, and the rear of the synovial capsule presents itself, and can be commodiously excised. In front, the incision falls in the center, between tibia and fibula. The extensor hallucis longus, and the large bunch of vessels and nerves, are drawn inwards with a flat hook. Now the capsula presented, and was excised. Dead cartilage was pulled out, and the result was splendid. If space should prove too small in some other case, the reporter suggests to lengthen the incision in the rear a little around the heel, to peel the musculo-cutaneous mass off a little towards both sides, and to saw a part of the heel off with a chain-saw, leaving it hang on the tendo Achillis. Thus a far greater access to the joint is necessarily secured without interfering with the vasa tibialia. After extirpation of the capsule, respectively gouging of talus or so is finished, the resected heel is to be nailed to its former place by an ivory nail.—MARCELL HARTWIG.

